# Short-Term Protein Supplementation Does Not Alter Energy Intake, Macronutrient Intake and Appetite in 50–75 Year Old Adults

**DOI:** 10.3390/nu13051711

**Published:** 2021-05-18

**Authors:** Esme R. Tuttiett, Dan J. Green, Emma J. Stevenson, Thomas R. Hill, Bernard M. Corfe, Elizabeth A. Williams

**Affiliations:** 1 Department of Oncology and Metabolism, The Medical School, The University of Sheffield, Sheffield S10 2RX, UK; Bernard.Corfe@newcastle.ac.uk (B.M.C.); e.a.williams@sheffield.ac.uk (E.A.W.); 2 School of Health and Related Research, Regent Court, The University of Sheffield, Sheffield S1 4AD, UK; d.j.green@sheffield.ac.uk; 3 Human Nutrition Research Centre, Population Health Sciences Institute, Faculty of Medical Sciences, Newcastle University, Newcastle NE2 4HH, UK; Emma.Stevenson@newcastle.ac.uk (E.J.S.); tom.hill@newcastle.ac.uk (T.R.H.)

**Keywords:** sarcopenia, protein, appetite, supplementation

## Abstract

Ageing is associated with a reduction in muscle mass and strength, termed sarcopenia. Dietary protein is important for the maintenance of muscle mass through the promotion of muscle protein synthesis. However, protein is also reported to be a highly satiating nutrient. This raises concerns that protein intake for musculoskeletal health reasons in older adults may exacerbate age-related decreased appetite and may result in reduced energy and nutrient intake. This study aimed to investigate the effect of short-term protein supplementation and its timing (morning vs. evening), on energy and nutrient intake and appetite measures in middle-older age adults. Twenty-four 50–75 year olds were recruited to a randomised cross-over trial. In phase 1 (pre-supplementation) participants completed a food diary and reported hunger and appetite on three alternate days. During the second and third phases, participants consumed a 20 g whey protein gel (78 mL/368 kJ), for four days, either in the morning (after breakfast) or the evening (before bed), whilst completing the same assessments as phase 1. No differences in dietary intakes of energy, macronutrients and micronutrients were recorded when comparing the pre-supplementation phase to the protein supplementation phases, irrespective of timing (excluding the contribution of the protein supplement itself). Similarly, no differences were observed in self-reported feelings of hunger and appetite. In conclusion, a 20 g/day whey protein supplement given outside of meal-times did not alter habitual dietary intakes, hunger or appetite in this middle-older age adult population in the short-term. This approach may be a useful strategy to increasing habitual protein intake in the middle-older age population.

## 1. Introduction

Sarcopenia is defined as “*a syndrome characterised by a progressive and general loss of muscle mass and strength as we age*” [1]. There is a need to develop interventions to prevent or delay the onset of sarcopenia, thereby promoting maintenance of quality of life and independence in susceptible individuals. One approach is through increasing the rate of muscle protein synthesis; either by oral nutritional supplementation with protein or amino acids [2], or through increased food-derived protein intake [3].

Dietary protein intake correlates positively with lean muscle mass in older adults [4,5] and provides a potential strategy to preserve muscle mass and maintain physical function [6]. The current UK Reference Nutrient Intake (RNI) for dietary protein intake is 0.75 g per kilogram body mass, per day [7]. However, this recommendation is age-agnostic, despite evidence that older adults are less responsive to anabolic stimuli [8] suggesting that older individuals have elevated protein requirements. The current literature recommendations suggest that per-meal thresholds of 25–30 g of protein should be achieved at multiple feeding occasions, distributed across the wake cycle, to promote muscle protein synthesis in the older population [9]. The PROT-AGE study group and the European Society for Clinical Nutrition and Metabolism (ESPEN) have recommended intakes of 1–1.2 g/kg/d for healthy older adults, up to 1.5 g/kg/d for older people with acute or chronic disease and up to 2 g/kg/d for malnourished older adults. It was recently shown that 36% of those aged 65–89 years failed to meet general UK guidelines for protein intakes with over 85% failing to meet the ESPEN protein recommendations for older adults [8].

There is a decline in hunger and appetite in older individuals, termed the anorexia of ageing [10]. Protein is considered to be the most satiating of all the macronutrients [11], and high protein diets are commonly reputed to benefit weight loss [12]. Weigle et al. demonstrated that a high protein diet led to decreases in self-reported levels of hunger and an increase in fullness compared to an isocaloric diet. [13]. A further study demonstrated that protein suppressed rated hunger, although it did not alter food intake over the subsequent 24 h [14]. Research in this area has often been conducted in relation to weight-loss, when appetite suppression is desirable. However, older adults (>70 years) are often excluded from trials of this type. Nonetheless, a recent systematic review and meta-analysis in older adults (mean (SD) age of 71 (3.8) years) conducted Ben-Harchache et al., 2020 [15] found that while acute protein supplementation interventions may lead to suppressions of self-reported appetite measures, neither acute or longitudinal protein supplementation interventions led to reductions in energy intakes, once the energy of the supplement was accounted for; a finding not observed in younger adults [16].

Identifying the optimum time to supplement with protein could prove advantageous, particularly if diurnal targeting of intake could minimise the impact on hunger and appetite. Protein intakes are often skewed to larger consumption later in the day [8,17]. Moreover, these trends are particularly evident in older individuals [18,19]. Elevation of morning protein consumption may therefore reduce variation in diurnal intake profiles, as long as morning supplementation does not modulate appetite and intake at subsequent eating occasions.

In view of the putative benefits of dietary protein supplementation for reducing muscle loss in middle-older age it is important to understand the impact of supplementation on dietary intake in this population. This cross-over study aimed to investigate the effect of protein supplementation taken in the morning or evening on the dietary intake and appetite of a group of middle-older aged adults.

## 2. Materials and Methods

### 2.1. Study Population and Ethics

Twenty-four, healthy, community-dwelling participants, aged 50–75 years were recruited from Sheffield, UK and surrounding areas between April–June 2019. Recruitment was via both an electronic and physical poster advertising campaign targeting university staff, local networks and community groups. Inclusion criteria were: adults aged 50–75 years old. Exclusion criteria were: a BMI of <18 or >30 kg/m^2^, the presence of kidney issues or diabetes, dietary restrictions or the anticipation of a disruption to their habitual diet, e.g., as a result of holiday. Furthermore participants needed to be able to respond to the recruitment campaign (via email) and have no known cognitive difficulties. Written informed consent for participation was collected during a one-to-one enrolment discussion prior to the study.

Ethical approval for this study was granted by the University of Sheffield’s Ethics Committee (ethical approval number: 024856). This trial was registered on the ISRCTN registry database (registry number: ISRCTN99945020).

### 2.2. Design and Intervention

**Figure 1:**
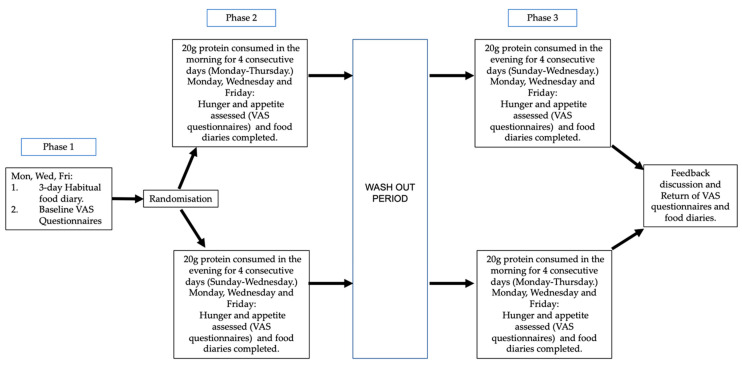
Graphical summary of trial workflow.

The study design was a randomised cross-over trial, which was comprised of 3 phases (Figure 1). Following recruitment of participants, height (cm) (Seca 217 portable height measure, model number: 217 1821 009) and body mass (kg) (Seca 761 floor scales, model number: 761 7019004) were measured. Participants were asked to record everything they ate or drank during a 24 h period, 3 times a week (Monday, Wednesday and Friday) using a physical food diary booklet. Participants were also provided with a food portion booklet, containing photographs from the Ministry of Agriculture, Food and Fisheries (MAFF) food atlas [20] to aid with their assessment of food portion sizes. Phase 1 (baseline) consisted of participants completing the 3-day food diary in addition to answering a validated visual analogue scale (VAS) appetite questionnaire, consisting of nine questions relating to feelings of hunger and appetite [21]. Participants were asked to complete the questionnaires three hours after the first meal of the day.

In the second and third phases, participants consumed a whey protein hydrolysate gel (containing 20 g protein (7 g of which are branched chain amino acids) and 376 kJ of energy) for 4 days, either in the evening (before bed) or in the morning (after breakfast) and completed the same tasks as phase 1. Morning protein supplementation required participants to consume the supplement 30–60 min after consumption of their first meal of the day, on Monday, Tuesday, Wednesday and Thursday. Evening supplementation took place 30–60 min before participants went to bed on Sunday, Monday, Tuesday and Wednesday. Thus, the timing of supplementation fell in line with the participant’s daily routine, and they were asked to note when they consumed this supplement in their study documentation. A 1-week wash-out period separated phase 2 and 3 before crossing over to the alternative time point. The protein supplement was a strawberry or lemon flavoured 78 mL gel (based on participant’s preference), containing 376 kilojoules of energy, 20 g of protein (whey), 1.8 g of carbohydrate (1.4 g of which sugars) and 0.1 g of fat (Science in Sport, Whey20, Nelson, Lancashire.)

All participants were given an identification code number, to keep their identity anonymous, and an online randomisation generator (http://www.randomization.com/, accessed: 15 April 2019) was used to assign participants to their study sequence. Stratified block randomisation, in block sizes of four, was utilised to ensure equal numbers of participants, in relation to their age category (50–64 years and 65–75 years), were receiving each treatment arm in all phases for the trial. Following completion of the trial, participants were invited to attend a verbal feedback session with the researcher. During this session food diaries and questionnaires were assessed for clarity and any anomalies or missing data were gathered. Compliance was also checked by participants completing a daily tick sheet, as part of their study documentation, to indicate if they had consumed their supplement.

### 2.3. Data Analysis

Dietary intake data from the food diaries was analysed using Dietplan7 nutritional analysis software (Forestfield Software Ltd., Horsham, UK). Protein intakes were also further analysed by adjusting for the participant’s body mass. This data was used to explore if participants were achieving recommended RNI for protein (0.75 g per kg body weight/d), in addition to higher values in line with literature recommendations for older adults to maintain physical function: 1–1.2 g per kg body weight/d for healthy older adults and 1.2–1.5 g per kg body weight/d for older adults with acute or chronic disease [8].

Histograms were investigated to examine normality in the continuous variables and were summarised using median/Inter-Quartile Range (IQR) due to the modest participant numbers. Categorical data were summarised with frequency and percentages. The Wilcoxon signed-rank test was used to explore differences between treatment arms: Baseline week (no protein supplementation) vs. morning supplementation vs. evening supplementation. A McNemars test was used to compare categoric paired data for information presented as percentages. Statistical significance was regarded as *p* < 0.05. All statistical analyses were undertaken in SPSS software (version 25).

## 3. Results

### 3.1. Participants

**Figure 2:**
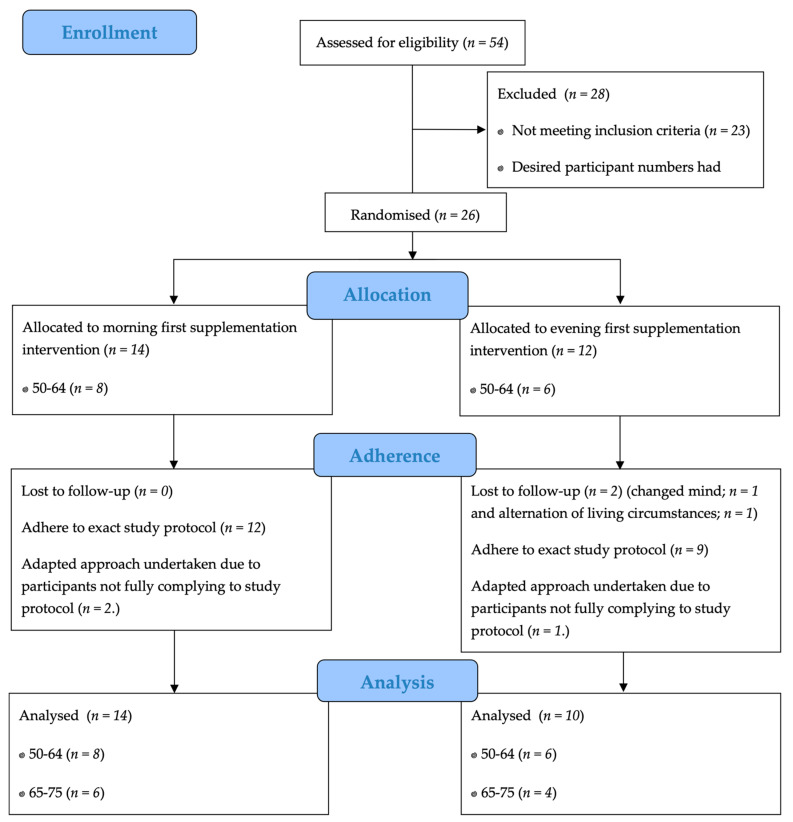
Consort flow diagram to show the enrolment process, allocation, adherence and final participant numbers included in the result analysis of the study.

Twenty-four participants were recruited to the study as shown in the CONSORT workflow (Figure 2). The majority of the participants were female (n = 20, 83%), their median age was 58 years (range 50–71) and they had a median (IQR) height, body mass and BMI of 166 (160.8–167.9) cm, 62.8 (59.1–75.5) kg, and 22.9 (21.8–26.6) kg/m^2^, respectively. Self-reported protein supplementation compliance was 100% for all participants.

### 3.2. Self-Reported Appetite Ratings

The impact of the protein supplement at different timings was compared with baseline measures of appetite. The responses to the VAS questionnaire are presented in Table 1. There were no differences in “hunger” (baseline vs. morning; *p* = 0.267, baseline vs. evening; *p* = 0.520); “satisfaction” (baseline vs. morning; *p* = 0.415, baseline vs. evening; *p* = 0.709); and “eating desire” (baseline vs. morning; *p* = 0.506, baseline vs. evening; *p* = 0.189.) Participants reported a significant reduction in the amount of food they thought they could eat (“intent to eat”) when the protein supplement was consumed irrespective of the time of supplemental protein consumption (baseline vs. morning; *p* = 0.012, baseline vs. evening; *p* = 0.035.).

### 3.3. Nutrient Intakes

Table 2 summarises energy and macronutrient intakes. The 20 g of additional whey protein received from the supplement during phases 2 and 3 is not incorporated in the analysis; all dietary data come from food/beverage consumption alone.

#### 3.3.1. Energy and Macronutrient Intake

Overall, there were no differences reported in energy intake. Similarly there were no differences observed in habitual protein, fat or carbohydrate intake at any stage of the trial, i.e., when the contribution made by the protein supplement was excluded from the analysis.

#### 3.3.2. Further Protein Analysis, Following Adjustments for Body Mass

Overall, 96%, 67% and 50% of participants were habitually consuming daily protein intakes of at least 0.75 g/kg BM, 1 g/kg BM, and 1.2 g/kg BM of protein, respectively, and the percentage of participants reaching these thresholds remained fairly consistent, irrespective of the phase of the trial (Table 2).

#### 3.3.3. Fibre and Micronutrient Intake

No differences from baseline were found for dietary fibre or micronutrient intakes, irrespective of the time of protein supplementation (all *p* > 0.143). This information can be found in the Supplementary Material (Figure S1).

## 4. Discussion

Increased dietary protein intake could alleviate loss of muscle mass and strength associated with ageing [4,5], but protein has satiating properties and could lead to reductions in total energy and nutrient intakes [12]. This study explored the impact of short term whey protein supplementation on subsequent hunger, appetite and dietary intakes in middle-older aged adults. The main finding of the research was that no differences in energy or nutrient intake provided by the habitual diet or hunger, and appetite ratings were associated with a 20 g daily protein supplementation.

These results are in agreement with the conclusion drawn by the recent analysis by (Ben-Harchache et al., 2020) [15] who found that protein supplementation led to, either a positive or no effect on total energy intakes, in both acute and longitudinal studies. Whilst some literature suggests that protein is satiating, [16] other studies have also found minimal effects on appetite of protein challenges in young, healthy individuals [22]. Likewise, other research studies have shown that protein supplementation did not reduce energy or macronutrient intakes [14,23]. The majority of the short-term and acute protein intervention trials require participants to consume the protein supplement as a pre-load. We chose a real-world analysis to explore the likely impacts of incorporating protein supplements on daily diet.

The low volume (78 mL) of the gel and its low energy (368 kJ) may minimise impact on hunger and fullness. Other benefits associated with using a gel was the ease of administration, allowing for precise control of protein intakes. There is considerable heterogeneity of protein supplementation strategies used in research, making consolidation of the literature in this field difficult. Consideration of how protein is consumed, including the form in which protein is delivered, for example bar, shake, gel or whole-food and the derivation of the protein source, including animal vs. plant-based protein, could be important in relation to appetite outcomes [19].

The rationale for choosing the two timepoints for our protein supplementation strategy are two-fold. Protein consumption is often suboptimal in the morning meal, and disproportionally skewed throughout the day to be greatest at lunch and dinner [17]. Post-breakfast offers a window of opportunity for introducing additional protein to support effective pulsing. In contrast evening supplementation may mitigate the enhanced muscle protein breakdown during sleep [24]. A randomised control cross-over trial by Morehen et al., 2020 [25] reported that a 40 g casein drink before bed did not affect ad libitum breakfast consumption or next-morning indices of appetite, energy intake or metabolism in older individuals in comparison to a maltodextrin or control beverage. In practice, when considering the impact of the supplement on diet and appetite, there were no differences in endpoints when comparing morning vs. evening protein supplementation. However, it is likely that by consuming an additional 20 g of protein/day, participants would achieve an overall intake 1–1.2 g/protein per kg body mass per day, in line with literature recommendations for older adults [8].

Rated appetite measurements were made to compare findings with acute studies. A limitation of the design was use of a single timepoint for rated appetite, 3 h after breakfast, as we anticipated this would be when hunger signals would be rising prior to lunch. The modest sample size means caution should exerted in interpretation. Despite that, Holt et al., 2017 [26], in an systematic review of studies using self-reported appetite scales, with average participant numbers of 30, suggested that participant numbers in this study are in-line with work conducted in this field.

### 4.1. Limitations

A technical limitation of the study is that it was not possible to include the 20 g of protein into this protein intake analysis, due to the study design, resulting in food diary records not corresponding directly with the evening protein supplementation. This study had a modest sample size and most of our participants achieved the UK’s protein RNI from their diet alone, in contrast with several studies of this age group [27]. Furthermore, the sample was predominantly female (83%), suggesting our sample may not be representative. Overall, this work supports the literature showing an absence of the acute effect of protein on appetite or energy intake, this time in a healthy, middle-age group.

### 4.2. Future Work

Longer-term intervention studies are needed to confirm these results, to explore the practicalities of prolonged protein supplementation, particularly in relation to appetite and energy intake impacts, in this age group and to investigate the impact of the supplement on muscle protein synthesis.

## 5. Conclusions

- There is negligible effect of protein supplementation on appetite and energy intake, in middle-older aged adults.
- Protein supplementation either in the evening or after breakfast may provide a viable strategy for increasing dietary protein intake in the middle-older age population.

## Figures and Tables

**Table 1:** Median (IQR) measured responses in mm to VAS questionnaires (0–100 mm) about self-reported feelings of hunger and appetite for all participants.

Self-Reported Measure	Participants (n = 24)				
	Baseline	Morning Supplementation		Evening Supplementation	
	Median (IQR) Response (mm)	Median (IQR) Response (mm)	*p*-Value ^$^	Median (IQR) Response (mm)	*p*-Value ^#^
**Hunger**	41 (29–48)	36 (16–48)	0.267	38 (24–44)	0.520
**Satisfaction**	53 (45–60)	52 (49–69)	0.415	50 (43–57)	0.709
**Intent to eat**	49 (43–58)	36 (26–51)	0.012 *	44 (35–54)	0.035
**Eating desire**	35 (23–58)	41 (22–49)	0.506	37 (21–46)	0.189

mm = millimetres. Hunger represents question 1 on the VAS (How hungry do you feel? 0 = I am not hungry at all, 100 = I have never been hungrier in my life.). Satisfaction represents question 2 on the VAS (How satisfied do you feel? 0 = How satisfied do you feel? 100 = I cannot eat another bite.). Intent to eat represents question 4 on the VAS (How much do you think you can eat? 0 = nothing at all, 100 = a lot). Eating desire represents question 5 on the VAS (How strong is your desire to eat (now)? 0 = Not at all, 100 = Very). Wilcoxon signed-rank *p*-values are baseline compared to invention phase; ^$^ represent baseline-morning and ^#^ denotes baseline-evening. * Significantly different from baseline, *p* < 0.05.

**Table 2:** Median (IQR) energy and macronutrient intakes of all participants across the different phases of the trial.

Intake	Baseline	Morning Supplementation		Evening Supplementation	
	Median (IQR)	Median (IQR)	*p*-Value ^$^	Median (IQR)	*p*-Value ^#^
**Energy (kJ/d)**	7804 (6868–9317)	7420 (6419–9093)	0.376	7712 (6303–9344)	0.338
**Carbohydrate (g/d)**	216.4 (177.6–248.0)	202.8 (177.2–246.1)	0.587	204.5 (168.7–259.7)	0.361
**% of energy from carbohydrate**	46.8	46.9	0.987	45.4	0.658
**Fat (g/d)**	75.2 (63.2–99.1)	73.4 (59.5–95.4)	0.407	76.7 (65.1–96.4)	0.597
**% of energy from fat**	37.6	37.2	0.638	38.5	0.779
**Protein (g/d)**	80.6 (62.6–97.4)	74.1 (70.0–91.0)	0.753	73.1 (64.8–93.1)	0.700
**% of energy from protein.**	16.7	17.5	0.224	17.4	0.475
**Protein after body mass adjustments (g/kg BM)**	1.21 (1.03–1.45)	1.14 (1.03–1.45)	0.876	1.20 (0.94–1.39)	0.957
**Percentage of participants consuming ≥ 0.75 g of protein per kg body mass. (%)**	95.8	95.8	>0.999	91.7	0.885
**Percentage of participants consuming ≥ 1 g of protein per kg body mass. (%)**	66.7	79.2	0.615	66.7	>0.999
**Percentage of participants consuming ≥ 1.2 g of protein per kg body mass. (%)**	50.0	45.8	>0.832	45.8	0.832

For macronutrient intakes, data are presented as median (IQR) or the percentage contribution of each macronutrient to overall energy intakes. kJ/day = kilojoules of energy per day. g/d = grams per day. g/kg BM = gram per kilogram body mass. The percentage (%) of participants consuming at least either 0.75 g, 1 g or 1.2 g per kg body of protein are also presented. *p*-values are baseline compared to invention phase; ^$^ represent baseline-morning and ^#^ denotes baseline-evening.

## Data Availability

It is the intention of the authors to be fully transparent with the data and therefore all raw data and additional materials will be made available, upon request.

## Supplementary Materials

The following are available online at https://www.mdpi.com/article/10.3390/nu13051711/s1, Figure S1; Supplementary material displaying Median (IQR) dietary fibre and micronutrient intakes of all participants, per day, across the different phases of the trial.
