# Supplementary material for: Short-Term Protein Supplementation Does Not Alter Energy Intake, Macronutrient Intake and Appetite in 50–75 Year Old Adults
Source: Nutrients. 2021 May 18;13(5):1711. doi: 10.3390/nu13051711 (PMC8157839; doi:10.3390/nu13051711)
Supplement: Supplementary file 1 [file nutrients-13-01711-s001.zip › nutrients-1180098-supplementary.pdf]

Nutrient	*Baseline*	*Morning Supplementation*		*Evening Supplementation*	
	Median Intakes (IQR)	*Median Intakes (IQR)*	*p-value$*	*Median Intakes (IQR)*	*p-value#*
**Dietary fibre (g)**	20.6 (16.7-30.0)	23.3 (16.4-26.0)	0.849	22.6 (14.0-26.3)	0.143
**Calcium (mg)**	1058.5 (813.3-1345.3)	1039.0 (916.0-1308.0)	0.368	953.0 (822.8-1145.0)	0.738
**Magnesium (mg)**	360.0 (320.5-449.3)	375.5 (295.5-424.0)	0.833	368.5 (291.3-434.5)	0.278
**Sodium (mg)**	1867.5 (1402.0-2183.3)	1882.5 (1374.3-2480.0)	0.459	1784.5 (1431.0-2179.3)	0.580
**Potassium (mg)**	3601.0 (3024.8-4216.3)	3444.5 (3058.0-3781.5)	0.924	3211.5 (2895.5-3605.5)	0.143
**Iron (mg)**	11.4 (8.0-13.8)	11.7 (8.4-14.9)	0.738	11.2 (9.7-14.1)	0.978
**Retinol (µg)**	334.5 (223.8-414.5)	274.0 (219.0-378.5)	0.360	287.5 (225.5-478.5)	0.752
**Carotene (µg)**	2316.0 (1218.0-3972.8)	2926.5 (1620.3-4792.3)	0.293	1507.5 (629.8-3850.0)	0.979
**Riboflavin (mg)**	2.02 (1.67-2.39)	2.12 (1.63-2.44)	0.803	1.82 (1.59-2.31)	0.710
**Vitamin B6 (mg)**	1.60 (1.32-1.75)	1.63 (1.38-1.78)	0.837	1.61 (1.35-1.87)	0.613
**Vitamin B12 (µg)**	5.80 (3.50-7.63)	5.20 (3.15-6.65)	0.721	5.45 (3.18-6.43)	0.344
**Vitamin C (mg)**	103.5 (86.0-124.0)	88.5 (76-114.8)	0.558	82.0 (58.5-125.5)	0.246
**Vitamin D (µg)**	2.79 (2.22-4.98)	3.17 (1.34-5.63)	0.525	2.56 (1.01-4.24)	0.543
**Vitamin E (mg)**	8.51 (6.70-11.41)	8.15 (6.86-12.44)	0.311	9.13 (6.35-11.84)	0.312

Figure S1; Supplementary material displaying Median (IQR) dietary fibre and micronutrient intakes of all participants, per day, across the different phases of the trial.
g=grams; mg=milligrams, µg=micrograms.
P values are baseline compared to invention phase; IQR: Inter-Quartile Range; $ represent baseline-morning and # denotes baseline-evening
